# Artificial intelligence and cloud based platform for fully automated PCI guidance from coronary angiography-study protocol

**DOI:** 10.1371/journal.pone.0274296

**Published:** 2022-09-09

**Authors:** Vlad Ploscaru, Nicoleta-Monica Popa-Fotea, Lucian Calmac, Lucian Mihai Itu, Cosmin Mihai, Vlad Bataila, Bogdan Dragoescu, Andrei Puiu, Cosmin Cojocaru, Minoiu Aurelian Costin, Alexandru Scafa-Udriste

**Affiliations:** 1 Department of Cardiology, Emergency Clinical Hospital, Bucharest, Romania; 2 Department Cardio-Thoracic 4, University of Medicine and Pharmacy “Carol Davila”, Bucharest, Romania; 3 Department of Image Fusion and Analytics, Siemens SRL, Brasov, Romania; 4 Department of Automation and Applied Informatics, Transylvania University of Brasov, Brasov, Romania; Baylor Scott and White, Texas A&M College of Medicine, UNITED STATES

## Abstract

Ischemic heart disease represent a heavy burden for the medical systems irrespective of the methods used for diagnosis and treatment of such patients in the daily medical routine. The present paper depicts the protocol of a study whose main aim is to develop, implement and test an artificial intelligence algorithm and cloud based platform for fully automated PCI guidance using coronary angiography images. We propose the utilisation of multiple artificial intelligence based models to produce three-dimensional coronary anatomy reconstruction and assess function- post-PCI FFR computation- for developing an extensive report describing and motivating the optimal PCI strategy selection. All the relevant artificial intelligence model outputs (anatomical and functional assessment–pre- and post-PCI) are presented to the clinician via a cloud platform, who can then take the utmost treatment decision. The physician will be provided with multiple scenarios and treatment possibilities for the same case allowing a real-time evaluation of the most appropriate PCI strategy planning and follow-up. The artificial intelligence algorithms and cloud based PCI selection workflow will be verified and validated in a pilot clinical study including subjects prospectively to compare the artificial intelligence services and results against annotations and invasive measurements.

## 1. Introduction

Ischemic heart disease represents a heterogeneous pathology with lack of a standardized method for treatment even in the presence of multiple modern imaging and hemodynamic assessment methods of coronary lesions requiring personalized approach in each case. Multivessel coronary artery disease defined by the presence of at least 50% diameter stenosis of two or more epicardial coronary arteries is associated by poor prognosis and higher mortality compared with single vessel disease. It is substantially proved and known that in acute coronary syndromes (ACS), there is a clear indication of revascularization of the culprit lesion [1], but debatable recommendations exist in the case of other non-culprit stenosis, concerning the modality of researching their significance or the appropriate time of investigation and/or revascularisation. Identifying those coronary lesions that could beneficiate from percutaneous coronary intervention (PCI) during the index hospitalization for ACS is in some cases challenging. Previous clinical guidelines indicated the revascularization of only the culprit vessel in stable patients with ST-elevation myocardial infarction (STEMI) [2], but a growing body of evidence, such as COMPLETE [3], CULPRIT-SHOCK [4] or PRAMI [5] suggests that complete revascularization is beneficial in STEMI without cardiogenic shock. Fractional flow reserve (FFR) is the gold standard for the assessment of the functional significance of a coronary stenosis. Despite its proven utility it is still underused in daily clinical scenarios due to several drawbacks, including the need of increased financial resources and time. Several studies have shown the benefit of FFR-guided PCI in the reduction of major adverse cardiovascular events [6, 7], albeit in recent years two studies have shown conflicting results. FUTURE trial pinpointed no superiority at least at one year, of FFR-guided treatment in multivessel coronary artery disease patients, while in FAME3 PCI was inferior on the overall population to coronary artery by-pass grafting among patients with angiographic three-vessel disease, but on the subgroup analysis including patients with low SYNTAX scores (0–22), PCI guided by FFR appeared more beneficial [7] Given the recent technological advances, methods for image-based functional assessment of coronary stenosis based on coronary angiography (CA) have been introduced and validated, e.g. image based FFR computation [8–10] Angio-derived FFR technology pre-stenting and after several virtual PCI scenarios is cost-reduced, faster and more convenient for the patient and interventionist as it allows the FFR calculation without any wire exchange and pressure determination. The assessment of post-stenting FFR is also noteworthy as multiple large observational studies and post-hoc analysis of randomized trials [11, 12] showed that post-PCI FFR value is independently predictive of long-term outcomes, suggesting its incorporation into routine work flow in those patients having undergone pre-PCI FFR as part of clinical decision process.

The present paper exposes the protocol of a study whose scope is to provide to the clinician a simplified path for decision making regarding coronary lesions, interventional or conservative, in combination with clinical judgement to obtain the best results in a cost-effective fashion. Integrating routine angiographic images in a cloud platform that will provide treatment recommendation may permit in the future the development of a standardized method of interventional treatment amongst all cardiovascular centres. There are few data in the literature addressing the integration of CA images in a cloud database with the possibility of obtaining numerous anatomical and hemodynamic information that can provide a better treatment method in terms of safety and outcomes for each individual case.

## 2. Materials and methods

The study whose protocol is presented below is monocentric taking place at Emergency Clinical Hospital, Bucharest, and was approved by the Institutional Review Board no. 4587/29.04.2020. The integration of data from past CA exams and invasive FFR measurements undergone at Emergency Clinical Hospital for the development of several deep learning based models for coronary anatomy and function assessment as cloud based platforms will be accomplished by researchers from Transylvania University and the department of image and fusion and analytics from Siemens, Brasov.

In the first part of the project, the study will collect retrospectively available data from ACS subjects evaluated at Emergency Clinical Hospital with X-ray CA, FFR pre- and post-stenting (if PCI deemed necessary) in order to develop artificial intelligence (AI) based algorithms for comprehensive real-time coronary anatomy assessment, post-PCI FFR computation and optimal PCI strategy selection (including 190 patients with ACS, 3000 coronary angiography acquisitions, 270 invasive pre-PCI and 100 invasive post-PCI FFR measurements). All data were fully anonymized before access and did not require an informed consent for the retrospective analysis of the CA images as the ethics committee waived the requirement for informed consent. Once the AI algorithms are generated (detection of catheter tip, total occlusions, coronary diffuse disease, and stenoses, as well as three dimensional reconstruction and post PCI FFR computation), a pilot clinical study will verify and validate these models including prospectively 50 patients with ACS, complex coronary artery disease and functionally significant stenoses that require PCI. The data will be used for the validation of the AI models, the optimal PCI strategy selection, and the cloud based solution generated in the first part of the project. Patients will be included in this pilot study only after signing an informed consent form, and, a set of initial inclusion and exclusion criteria are checked. The inclusion criteria are as follows: acute coronary syndromes [13] with at least one lesion with visually estimated diameter stenosis ≥40% on CA and with the technical possibility to perform FFR in all lesions in subjects with life expectancy of at least one year. The exclusion criteria are: glomerular filtration rate <30 ml/min/1.73 m^2^ or the indication of surgical revascularization of the coronary lesions. The baseline evaluation at enrolment will include CA with Quantitative Coronary Analysis calculation, FFR and PCI (if deemed required).

### 2.1 Coronary angiography exams collection, anonymization and annotation

First, patient data is acquired by performing standard CA following the indications of the European Association of Percutaneous Cardiovascular Interventions for standard angiographic views and data is uploaded to the cloud. After acquisition, CA exams data is anonymized and stored, and then in detail annotated as follows: distal points of interest on main and side branches, catheter, total occlusions, diffuse disease, thrombus, non-culprit lesion identification and assessment of visual stenosis degree, start and end locations, correspondence between multiple CA sequences, and optimal PCI strategy selection. The CA data is then converted using the cloud based application where several AI based algorithms are run to process and assess the coronary anatomy and function, to produce an extensive report describing and motivating the optimal PCI strategy. All the relevant AI model outputs (anatomical and functional assessment–pre- and post-PCI) are presented to the clinician via the cloud platform, who can then take the optimal treatment decision.

**Fig 1:**
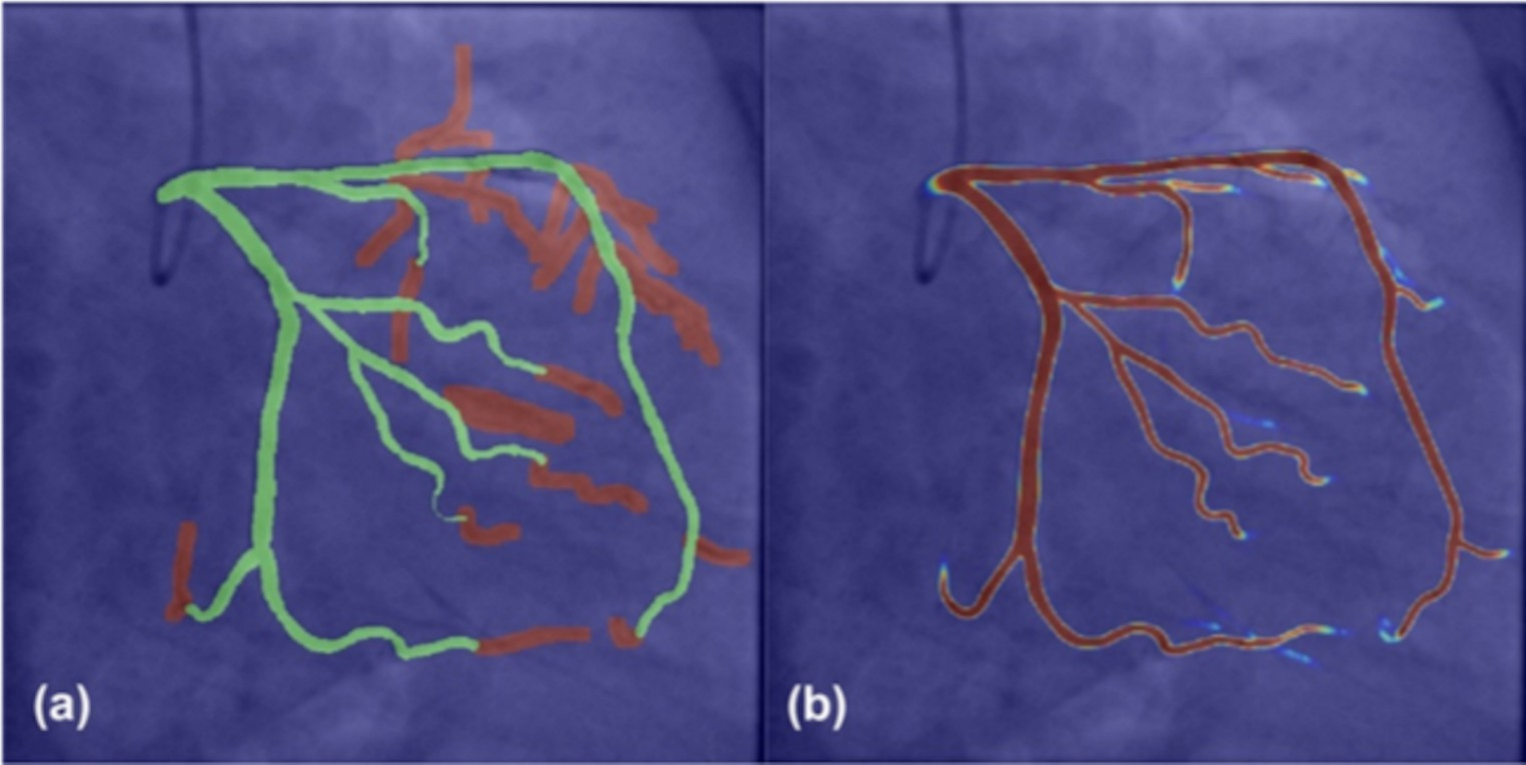
(a) Annotations of large (green) and small vessels (red), (b) Overlay of the input frame and the predicted probability map.

Regarding the development of deep learning models for real-time coronary anatomy assessment from CA exams, we will first focus on developing a methodology for filtering the DICOMs of the CA exams. On average each CA exam consists of 4–30 individual angiographic acquisitions, but not all of these are useful, since, in some of them no contrast agent is used, have a very small number of frames or inadequate radiologic regime. At this stage we will rely on two previously developed AI based models: vessel model (Fig 1) identifying the acquisitions with properly visible coronary arteries or left/ right coronary artery view classification, that recognizes the coronary tree visible in the coronary angiography image (left coronary artery/right coronary artery). Other filtering conditions that will be employed are the presence of ECG signal or a set of pre-defined DICOM tags (e.g. frame rate, image size).

### 2.2. Artificial intelligence algorithms for the detection of catheter tip, total occlusions and coronary diffuse disease

**Fig 2:**
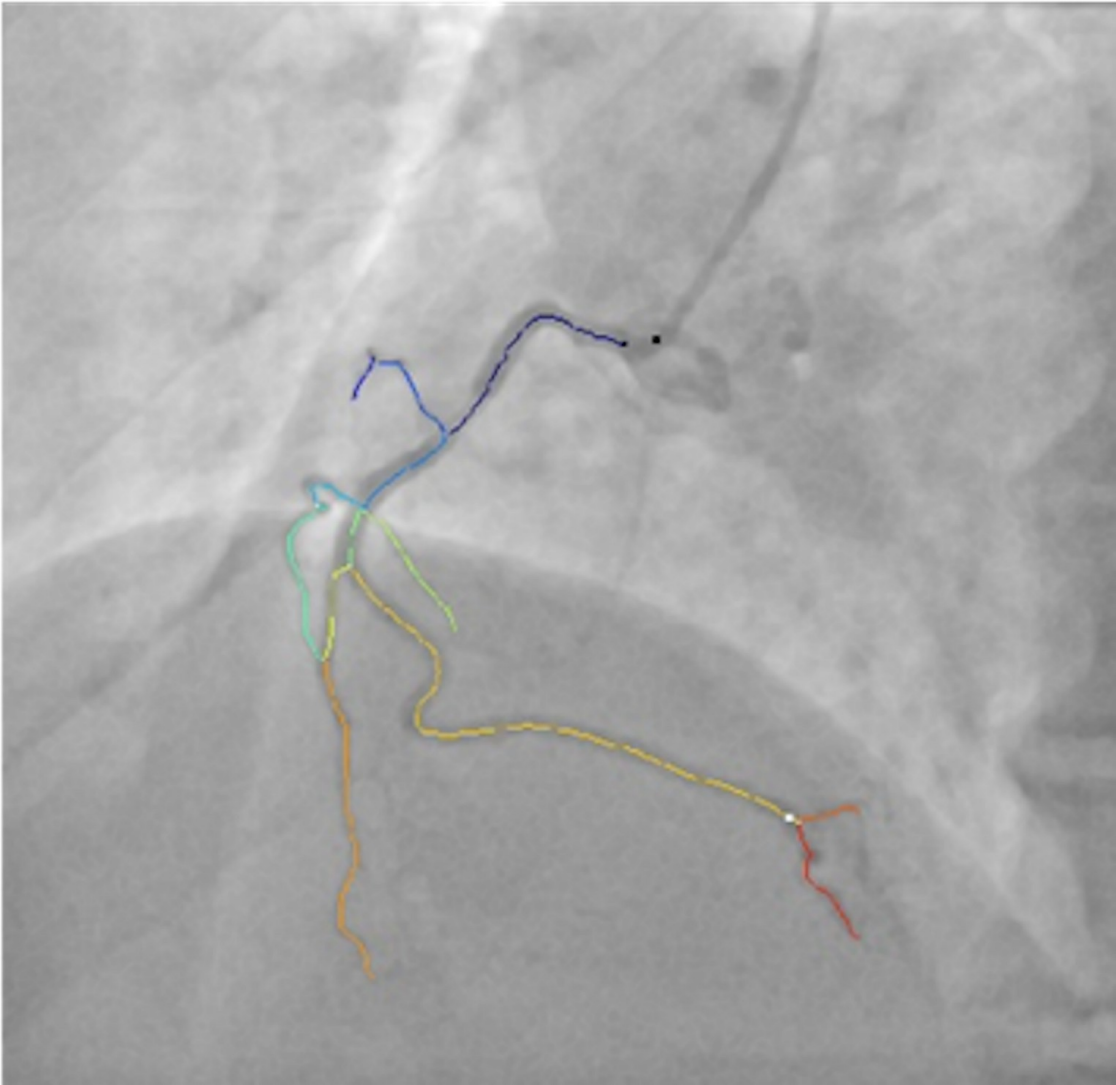
Sample envisaged detection of the distal point (white circle) for a right coronary artery image. The black circles the catheter tip.

Additionally, we will focus also on developing an AI improved model in terms of the speed of the processing units or the a priori knowledge of the number of catheters [14, 15], for detecting the catheter, the goal being to identify the tip of the catheter, which is typically placed in the coronary ostium. Thus, the catheter tip can be further employed as a surrogate for the coronary ostium. Different problem formulations will be considered for identifying the catheter tip such as localization problem (regressing the catheter tip coordinates), binary segmentation problem (placing a circle with a fixed diameter at the catheter tip), segmenting the entire catheter and performing a post-processing operation to identify the catheter tip. Secondly, we will develop an approach for identifying all distal points of interest. A set of rules will be defined for distal point identification (Fig 2): one distal point for each main branch (left anterior descending, circumflex, right coronary artery) with a diameter at this level of at least 1 mm, one distal point for each side branch with a diameter of at least 1 mm and at least one coronary stenosis with a stenosis degree > 30%.

The correct identification of the catheter tip and distal points is crucial for other tasks such as lumen segmentation, reconstruction, stenosis detection, hemodynamic assessment, and wall shear stress computation.

Two other activities, similar to each other, focus on the AI based detection of total occlusions and diffuse disease. These aspects are important for being able to correctly compute virtual FFR pre- and post-PCI. The problem formulation defined above for the catheter tip detection will also be considered within these tasks. Given that the tasks are correlated, we propose to use a multi-task deep learning approach. Deep learning is part of a broader family of machine learning algorithms based on artificial neural networks with representation learning. Learning can be supervised, semi-supervised, or unsupervised [16]. Multi-task learning refers to the setting in which multiple learning tasks are solved in an application, while exploiting commonalities and differences across tasks. Multi-task learning can be performed in several ways:

- hard parameters sharing: a single deep neural network is employed, where the first part of the network is applied for all tasks, and then one specific head is used for each task
- soft parameter sharing: separate deep neural networks are employed for each task, having a similar structure, i.e. the dep neural network layers are constrained to me similar
- cascaded models: the models are called sequentially, i.e. a first model takes the input data and performs a prediction, the second model takes the same input, and additionally the output of the first model to perform the prediction, etc.

### 2.3 Artificial intelligence algorithms for three dimensional reconstruction, stenosis detection and post-PCI FFR computation

Next, we will work on developing an AI based algorithm for comprehensive three dimensional (3D) reconstruction from multiple angiographic acquisitions. A basic version of a 3D reconstruction algorithm has been also developed during a previous project, HEART project [17], in which given two X-ray angiography images providing different views for the same coronary artery tree, with at least 30 degrees angular separation between them, and the segmentation of the vessels of interest in both images, the algorithm returns the position and shape in 3D space of the vessels of interest.

However, this methodology allows only for the reconstruction of a part of the coronary tree. To reconstruct the entire arterial tree, one needs to use all available angiographic acquisitions, rebuild parts of the coronary tree, and then stitch them together to obtain the full tree. Such an approach is required since CA images are two dimensional projections, and certain segments may not be well visible due to overlap or foreshortening. Two steps are critical for this activity.

The first one refers to deep learning based landmark detection. We propose to use an end-to-end trainable, fully convolutional network to detect the salient landmarks (vessel bifurcations) existing in angiography images. Bifurcations are the intrinsic landmarks that define the geometry of the underlying vessel tree, and most of them are co-existing across views, thus could be utilized as evidence to construct correspondence across views. The second step performs an ancestry-respectful optimal matching. First, the start and end points are defined for the segment of interest (the seed points): catheter tip and distal point(s) of interest. Given the seed points, the detected bifurcation points from the previous step, we construct a chain of points (called tree), representing the points from root to leaf of the branch, at this point obtaining multiple trees, one per angiographic acquisition. We define the matching of detected landmarks as a non-surjective injection function that maps pairs of detected landmarks across views. Also within this step, stenoses are detected based on the vessel lumen segmentation and the centerline detection, for which algorithms are already available from previous projects [18]. The stenosis detection algorithm uses as input the diameter information at each location and identifies all stenoses based on the radius profile along the centerlines.

**Fig 3:**
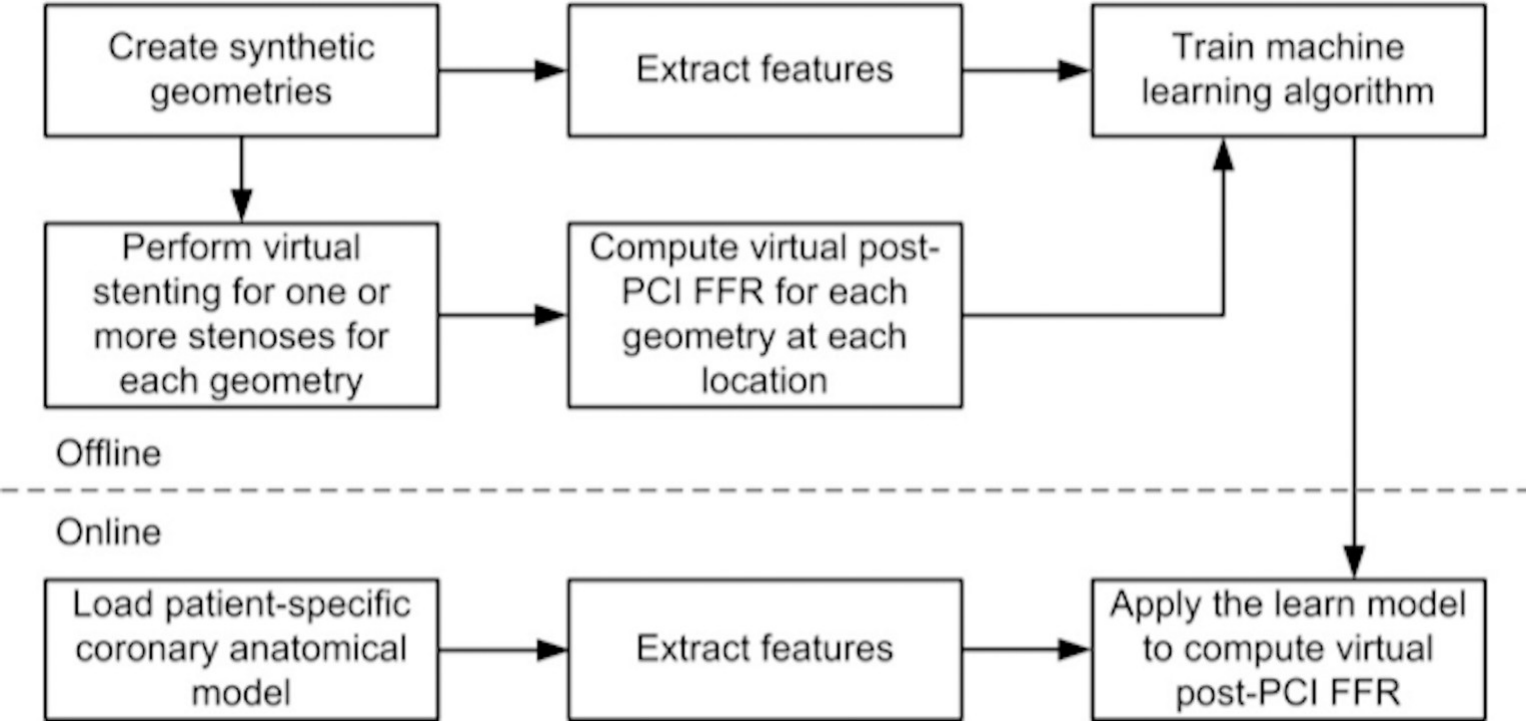
Approach for artificial intelligence based virtual post-percutaneous coronary intervention fractional flow reserve (FFR) computation.

Also an AI based algorithm for real time post-PCI FFR computation will be developed. Using a computational fluid dynamics based approach for post-PCI FFR computation introduced in a previous project-HEART [17], for which a priori virtual stenting procedure was required, herein we will develop a novel approach capable of producing results in real time, which takes as input the pre-PCI anatomical information, stent information (size, length, location) of the coronary arteries, outputting directly the virtual post-PCI FFR values at all locations of the coronary tree. The AI model will be developed using solely synthetic data (Fig 3), similar to a strategy adopted in a past project [10]. The invasively measured post-PCI FFR values will be used only for validating the model.

### 2.4. Artificial intelligence based algorithms as cloud services and development of a web client application

Further on, a methodology for optimal PCI strategy selection will be developed relying heavily on the previously developed models for 3D anatomical model reconstruction, stenosis detection and virtual post-PCI FFR computation. Specifically, once the 3D anatomical model is available, and all stenoses have been detected (including diffuse disease), the AI models for wall shear stress (WSS) [19] and virtual FFR calculation will be run to evaluate the results of all possible combinations of PCI procedures for the identified stenoses. A very large number of combinations will be obtained using also different stent configurations (e.g. length/size). This is why it is crucial to rely on AI based models capable of producing results in real time, instead of computational fluid dynamics based models which are computationally intensive. A fully automated optimization based approach will be defined, relying on a cost function computed from the pre- and post-PCI WSS and FFR values. The goal of the cost function based optimization will be to obtain virtual post-PCI FFR values that are close to one, while limiting the number of stents, and also to normalize the WSS values in the entire coronary tree. The optimal PCI strategies will be validated and compared against those generated during the annotations on the CA database.

A few remarks are to be made which are valid for all activities employing deep learning-based approaches. First of all, the typical training–validation–test split of 70% - 15% - 15% will be employed. During training, for an ideal training–validation–test split a clustering algorithm is applied at patient level ensuring that a balanced split is performed, i.e. all annotations are present with a similar density in the training, validation, and test data sets. For an optimal result, different clustering algorithms will be considered: k-means, mean shift, mini batch K-means, spectral clustering, agglomerative clustering, Birch, DBSCAN, affinity propagation, etc.

**Fig 4:**
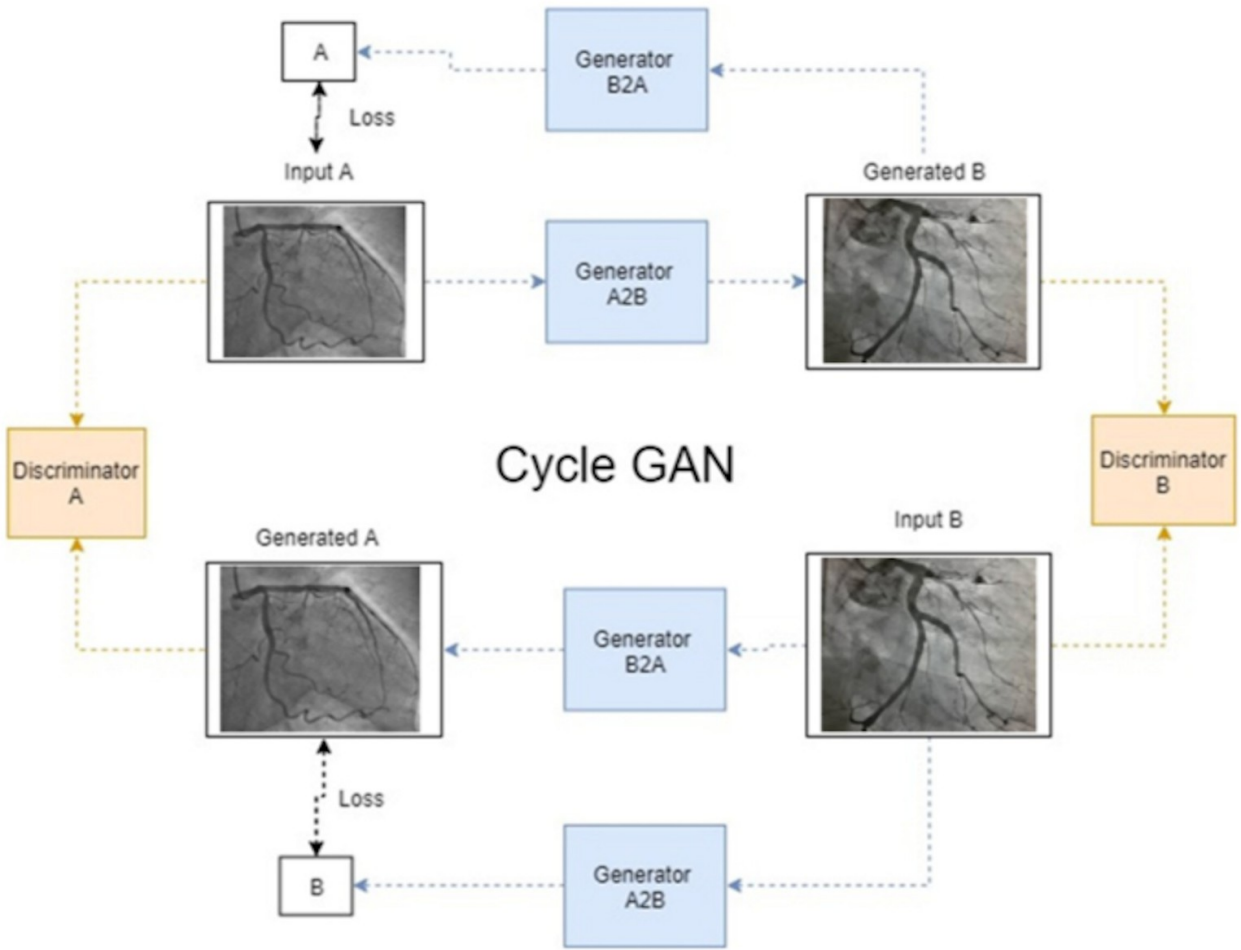
Cycle GAN approach for performing style transfer between healthy and pathological coronary angiography images slices.

Given that the retrospectively available patient database is large, enough data should be available to train accurate predictors. However, some of the aspects that are being followed within the activities described above, e.g., total occlusion, may have a low prevalence. On the other side, large training datasets are required for training highly accurate deep learning models. Thus, once all datasets will have been annotated, we will perform a careful analysis of the prevalence of all aspects of interest. If a low prevalence is found, other techniques for generating additional synthetic data will be employed. Specifically, healthy coronary angiography acquisitions will be selected and perform a style transfer using a cycle GAN approach [20], to generate pathological CA images. The proposed architecture is displayed in Fig 4, where two domains are considered: healthy CA acquisitions (domain A) and pathological CA acquisitions (domain B). The goal of cycle GAN is to transfer the style both from domain A to B, and from B to A. The loss function is calculated between the results and the original coronary angiography images.

Overall the usage of synthetic data provides several advantages such as: a very large number of cases that can be automatically generated, leading thus to an extensive database, rare pathological cases better sampled and complex pathological configurations generation, since that synthetic data can be completely automated with a reduced cost. In order to optimize the accuracy of the models, a specialized framework will be employed for hyperparameter tuning, e.g. Automated machine learning (AutoML), Bayesian Tuning and Bandits (BTB), etc.

**Fig 5:**
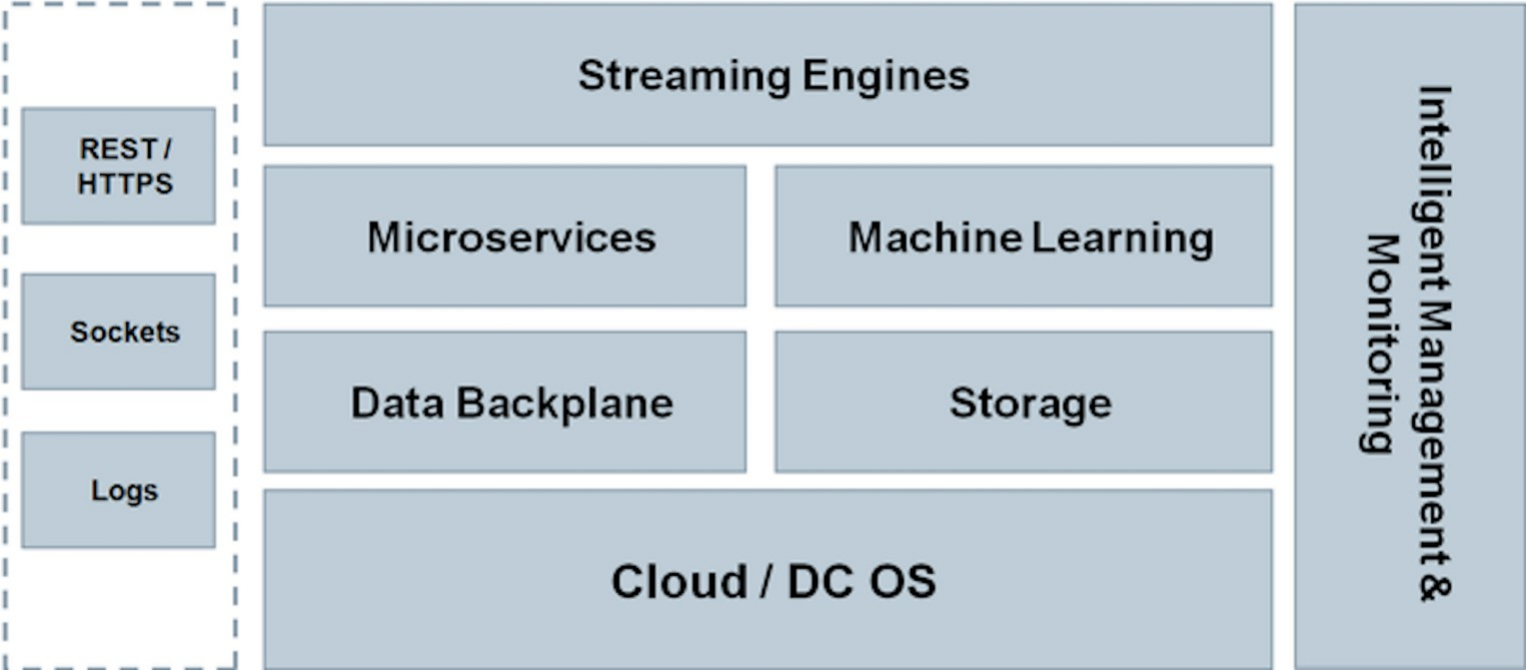
Overall concept and specific components for developing the cloud based solution.

In the end, a cloud based solution will be developed specifically addressing user management (authentication, authorization—data access control through a system of roles, groups and permissions), data handling, fast data processing–components that need to react as soon as data becomes available, zero-foot print applications that allow data visualization and data insight (available on PC, tablet and phone), and fault tolerant services. Fig 5 displays the overall concept and the specific components for developing the cloud based solution.

**Fig 6:**
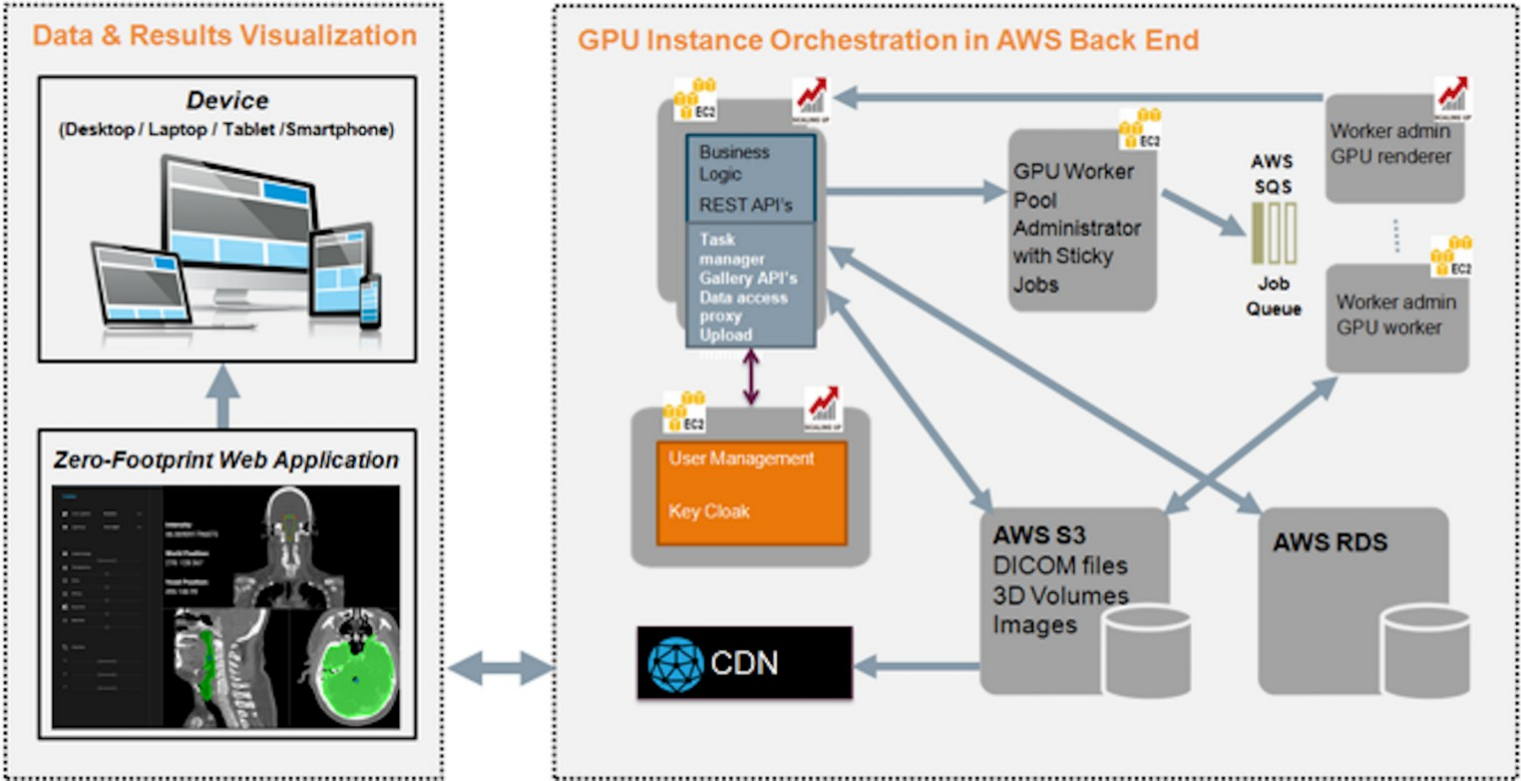
Graphic processing unit instance orchestration on the cloud.

Since many of the advanced analytics tools are run on massively parallel processors (graphics cards) we will develop a methodology for graphic processing unit instance orchestration on the cloud (Fig 6). The actual implementation will be based on Amazon Web Services, however achieving cloud agnosticism will be a major target during the development stages.

In the end, the AI algorithms and cloud based PCI strategy selection workflow will be verified and validated in a small scale prospective clinical study as mentioned previously. The CA data from this study will be processed using the cloud based application, and AI services and results are compared against the annotations and invasive measurements.

## 3. Discussion

Previously reported researches for automated analysis of CA images have addressed certain topics like segmentation, reconstruction and/or non-invasive functional assessment. However, few solution has been reported until now on reduced sample sizes, capable of performing a comprehensive analysis of the coronary arteries from CA data alone for optimal PCI strategy guidance [21–23].

Artificial neural networks based models with utility in interventional cardiology are in continuous development, either predicting PCI-related complications [24], or showing non-inferiority in the interpretation of instantaneous wave free pressure-wire pull back data compared to expert humans in determining the hemodynamic appropriateness for PCI [25].

The present study whose protocol was presented above proposes a novel solution, based on cascade deep learning based models for a comprehensive and fully automated CA based coronary analysis. The processing pipeline will be made available as a cloud based application, which can be included in the daily practice of cardiac catheterization laboratories enabling a personalized optimal decision in coronary artery disease patients.

The model proposed will assure a better planning of interventions along with an increased precision of procedures by prediction of final results and decrease of inter and intraoperator variability for an improved catheterization lab workflow. Although, the real impact of this AI cloud based platform for fully automated PCI guidance needs to be investigated in future, larger, prospective studies. In this perspective, after the validation of the platform in the pilot-phase of the study, we aim to investigate its clinical impact comparing a cohort of roughly 200 patients presenting with ACS without cardiogenic shock, and displaying other coronary lesions potentially significant apart of the culprit stenosis, in which the revascularization is done with the AI algorithms with a cohort in which the revascularization is conducted without the aid of the platform. The primary end-points will be a composite of death, myocardial infarction or urgent revascularization, while secondary outcomes will be the individual components of primary end-points, non-urgent revascularization, cardiac death, angina class and quality-of-life. The attempt of the cloud database will be to stratify patients with complex anatomies after ACS that would beneficiate more from medical treatment or from revascularization during the index hospitalization or during another scheduled admission. Another facet to be considered is the cost-efficiency of the method proposed, that is supposed to significantly reduce the financial burden produced by hospitalization for urgent revascularization or hospital outpatient consultations for angina.

## 4. Conclusions

By integrating medical data in a cloud database with AI algorithms capable of multivariable information processing and elaboration of instant treatment recommendation, there is a solid foundation for future developments in cardiovascular or other medical fields, simplifying the path towards standardization of medical treatment in general and interventional revascularization in particular. The present article presents the protocol of a study aiming to develop a cloud based application which can be integrated directly in the clinical settings of interventional cardiology departments providing coronary stent therapy guidance enabling a real time evaluation of the optimal PCI strategy.
